# Construction and validation of a risk prediction model for aromatase inhibitor-associated bone loss

**DOI:** 10.3389/fonc.2023.1182792

**Published:** 2023-04-27

**Authors:** Meiling Chu, Yue Zhou, Yulian Yin, Lan Jin, Hongfeng Chen, Tian Meng, Binjun He, Jingjing Wu, Meina Ye

**Affiliations:** Department of Breast Surgery, Longhua Hospital Shanghai University of Traditional Chinese Medicine, Shanghai, China

**Keywords:** aromatase inhibitors, breast cancer, bone loss, risk prediction model, XGBoost, logistic regression, LASSO regression

## Abstract

**Purpose:**

To establish a high-risk prediction model for aromatase inhibitor-associated bone loss (AIBL) in patients with hormone receptor-positive breast cancer.

**Methods:**

The study included breast cancer patients who received aromatase inhibitor (AI) treatment. Univariate analysis was performed to identify risk factors associated with AIBL. The dataset was randomly divided into a training set (70%) and a test set (30%). The identified risk factors were used to construct a prediction model using the eXtreme gradient boosting (XGBoost) machine learning method. Logistic regression and least absolute shrinkage and selection operator (LASSO) regression methods were used for comparison. The area under the receiver operating characteristic curve (AUC) was used to evaluate the performance of the model in the test dataset.

**Results:**

A total of 113 subjects were included in the study. Duration of breast cancer, duration of aromatase inhibitor therapy, hip fracture index, major osteoporotic fracture index, prolactin (PRL), and osteocalcin (OC) were found to be independent risk factors for AIBL (*p* < 0.05). The XGBoost model had a higher AUC compared to the logistic model and LASSO model (0.761 *vs.* 0.716, 0.691).

**Conclusion:**

The XGBoost model outperformed the logistic and LASSO models in predicting the occurrence of AIBL in patients with hormone receptor-positive breast cancer receiving aromatase inhibitors.

## Introduction

According to statistics from the International Agency for Research on Cancer, breast cancer is the most common malignant tumor worldwide, accounting for 11.7% of new cases in 2020 (1). Among breast cancer subtypes, hormone receptor-positive breast cancer represents approximately 70% (2). Endocrine therapy is an effective approach to reducing estrogen secretion, which can lower the risk of recurrence and metastasis by up to 50% (3). Aromatase inhibitors (AIs) are the primary drugs used for endocrine therapy in postmenopausal patients with hormone receptor-positive breast cancer. Ovarian function suppression (OFS) and AIs are also used in combination to treat premenopausal patients. AIs can improve the prognosis of hormone receptor-positive breast cancer (4), but the continuous reduction of estrogen levels can lead to aromatase inhibitor-associated bone loss (AIBL), which is associated with bone metabolic dysfunction, arthralgia, osteopenia, and osteoporosis (5). Logistic regression is a variant of the generalized linear model (GLM) commonly used for binary classification problems (6), while least absolute shrinkage and selection operator (LASSO) regression is used to select feature variables that are most useful for the model to avoid overfitting. Cross-validation is usually required to determine the best regularization parameters for LASSO regression (7). In contrast to traditional linear regression, eXtreme gradient boosting (XGBoost) is a machine learning algorithm that uses decision tree ensembles to build predictive models. It calculates the importance of features to eliminate unnecessary features and improve model performance and interpretability (8). The study established and verified XGBoost, LASSO regression, and logistic regression models to predict the incidence of AIBL. The prediction efficiency of the three models was compared, and the model with the best performance could provide valuable insights for AIBL treatment and prevention.

### Study design

**Figure 1:**
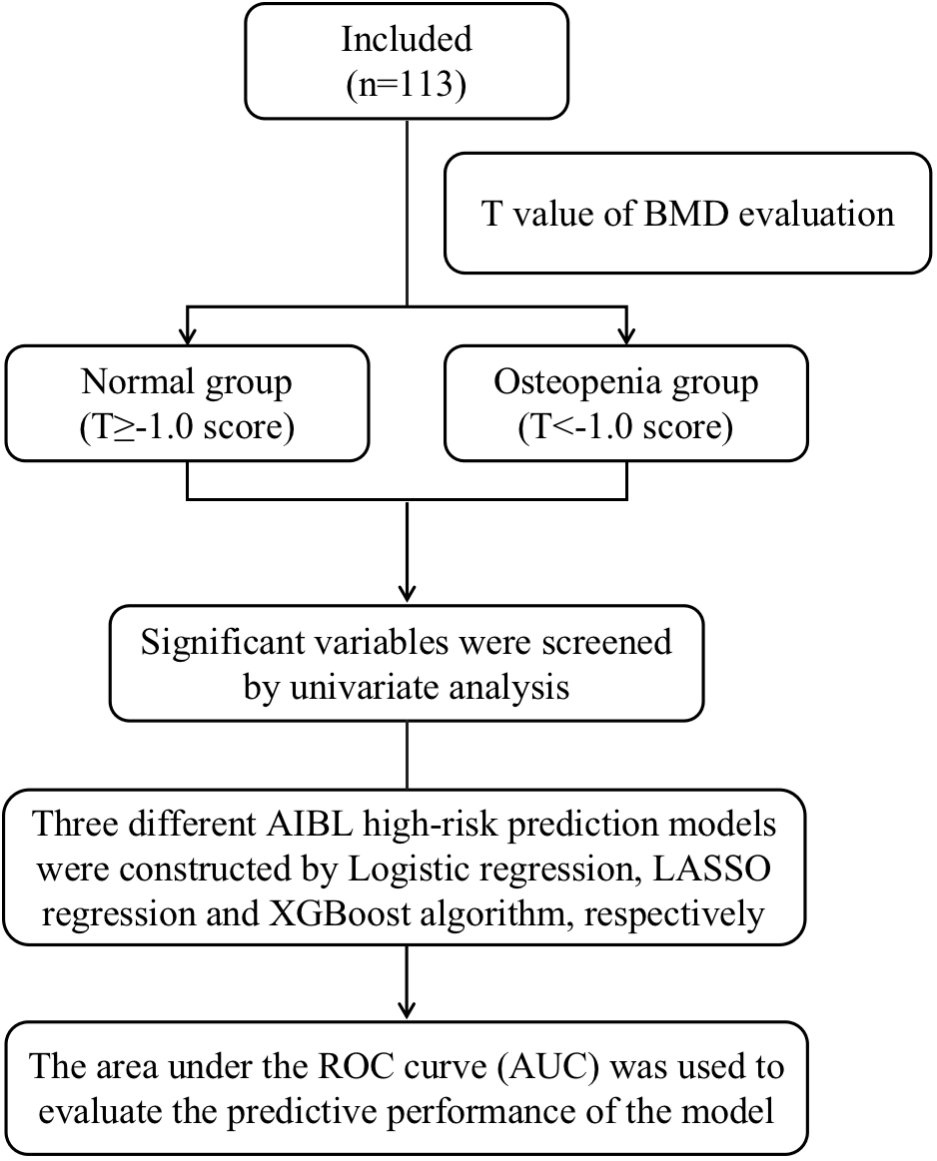
Schematic diagram of the research flowchart.

This study is a prospective interventional single-center study. The subjects of this study were breast cancer patients who were treated in the first department of the breast cancer clinic of Longhua Hospital affiliated with Shanghai University of Traditional Chinese Medicine from February 2022 to March 2023. A schematic diagram of the research flowchart is shown in **Figure 1**.

The Medical Ethics Committee of Longhua Hospital affiliated with the Shanghai University of Traditional Chinese Medicine approved this research. Written informed consent was obtained from all individual participants included in the study. The clinical trial registration number is ChiCTR2200057785.

### Participant

#### Diagnostic criteria

The breast cancer diagnosis was based on the 2022 Guidelines for Diagnosis and Treatment of Breast Cancer by the Chinese Society of Clinical Oncology (CSCO), confirmed by basic and molecular pathology. Osteoporosis diagnosis was based on the bone mineral density measured by dual-energy X-ray absorptiometry (DXA) according to the World Health Organization (WHO) 1994 criteria (normal, T score ≥ −1.0; osteopenia, −2.5 < T score < −1.0; osteoporosis, T score ≤ −2.5) (9).

#### Inclusion criteria

1) Female patients diagnosed with breast cancer, with positive estrogen receptor (ER) and/or progesterone receptor (PR) by immunohistochemical examination of postoperative pathology; 2) age between 18 and 65 years; 3) patients who have undergone surgery, chemotherapy, radiotherapy, and/or targeted therapy; 4) patients who have received aromatase inhibitor treatment for at least 2 months; 5) patients who voluntarily underwent clinical investigation and related examinations and provided signed informed consent.

#### Exclusion criteria

1) Patients with concurrent diseases affecting bone metabolism (such as Cushing’s syndrome, hyperthyroidism, rheumatism, or rheumatoid arthritis); 2) patients who have used hormone replacement therapy (such as glucocorticoids, parathyroid hormone, and estrogen) within 6 months; 3) patients with serious primary diseases of the cardiovascular, hepatic, renal, and hematopoietic systems; 4) patients with cognitive impairment and psychiatric disorders.

## Materials and methods

### Observation indexes

1) Bone mineral density (BMD) was measured by dual-energy X-ray absorptiometry (DXA).
2) Patient baseline characteristics, breast cancer diagnosis, and treatment information were collected as part of the study.
3) Three questionnaires were used in the study.
1) The Fracture Risk Assessment Tool (FRAX) measures the probability of major osteoporotic fracture and the 10-year probability of hip fracture due to osteoporosis. The FRAX scale was used to evaluate fracture risk in breast cancer patients receiving AI treatment, but BMD values were also included in the assessment to improve the accuracy of the algorithm (10). However, it has also been suggested that FRAX may underestimate the 10-year fracture risk in breast cancer patients (11).
2) The Western Ontario and McMaster Universities Osteoarthritis Index (WOMAC) is a commonly used scale to evaluate the severity of hip and knee arthritis, including pain, stiffness, and physical function (12).
3) Modified Score for the Assessment of Chronic Rheumatoid Affections of the Hands (M-SACRAH) is primarily used to assess functional status, stiffness, and pain in patients with hand osteoarthritis and rheumatoid arthritis (13). It has been widely used to assess the severity of osteoarticular side effects in patients undergoing AI treatment (14).
4) Examination indicators: the laboratory blood tests included routine biochemical tests and daily monitoring of endocrine therapy for breast cancer patients. Blood samples were collected from all subjects in the fasting state, and all subjects had completed surgery, chemotherapy, radiotherapy, and targeted therapy.

### Data management

All data were collected using one-to-one questionnaires completed by the researcher and research subjects within 20 min to ensure accuracy. The collected data were then entered into an Excel sheet within 1 week of survey completion. Additionally, 20% of the data were manually checked for input errors and corrected accordingly.

### Statistical methods

Descriptive statistics, including mean ± standard deviation for continuous variables and a number of cases and percentages for categorical variables, were used to describe the data. The independent sample *t*-test was used for comparing continuous variables with normal distribution between two groups, and the chi-square test was used for comparing categorical variables between groups. Statistical analyses were performed using SPSS 26.0 software.

Univariate analysis was performed to identify significant factors, followed by XGBoost machine learning, LASSO regression, and logistic regression analysis to build a prediction model to evaluate the association between the clinical characteristics of the study population, blood test indicators, and the incidence of AIBL. This analysis was conducted using R4.2.2, with statistical significance set at *p* < 0.05.

## Results

### Inclusion of patient

All eligible participants who voluntarily enrolled in the study completed the epidemiological questionnaire and underwent blood index detection and BMD examination. A total of 113 project volunteers were recruited from February 2022 to March 2023. Among them, 66 patients had osteopenia or osteoporosis, while the remainder had normal bone mass.

### Univariate analysis

After normal distribution tests and intergroup comparison analyses were performed, the following screening indicators with significant differences between the osteopenia and normal bone mass groups were obtained: duration of breast cancer (*p* = 0.001), duration of aromatase inhibitor treatment (*p* = 0.002), major osteoporotic fracture index (*p* < 0.001), hip fracture index (*p* < 0.001), prolactin (PRL) (*p* = 0.012), and osteocalcin (OC) (*p* = 0.025). These findings are presented in **Tables 1**, **2**.

**Table 1:** Results of epidemiological univariate analysis in normal and osteopenia groups.

Variables		Osteopenia group (n = 66)	Normal group (n = 47)	*p*-Value (2-sided)
Age		48.61 ± 8.79	46.11 ± 7.50	0.113
BMI		22.87 ± 2.92	23.31 ± 2.70	0.418
Education	High school and below	19 (28.8%)	9 (19.1%)	0.242
	More than university	47 (71.2%)	38 (80.9%)	
Age at menarche		13.92 ± 1.13	13.60 ± 0.99	0.073
Menopause	No	40 (60.6%)	34 (72.3%)	0.265
	Less than 10 years	18 (27.3%)	11 (23.4%)	
	More than 10 years	8 (12.1%)	2 (4.3%)	
Number of pregnancies	No	1 (1.5%)	5 (10.6%)	0.153
	Once	24 (36.4%)	18 (38.3%)	
	Twice	22 (33.3%)	11 (23.4%)	
	More than three times	19 (28.8%)	13 (27.7%)	
Number of production times	No	3 (4.5%)	5 (10.6%)	0.391
	Once	48 (72.7%)	34 (72.3%)	
	Twice	15 (22.7%)	8 (17%)	
Family history of cancer		20 (30.3%)	15 (31.9%)	0.855
Duration of breast cancer (months)		27.08 ± 12.57	19.09 ± 10.74	0.001
Lymph node metastasis		27 (40.9%)	24 (51.1%)	0.285
Anthracycline-based chemotherapy		32 (68.1%)	15 (31.9%)	0.078
Targeted chemotherapy		11 (16.7%)	7 (14.9%)	0.800
Radiation therapy		47 (71.2%)	36 (76.6%)	0.523
Duration of aromatase inhibitor therapy (months)		19.92 ± 11.95	13.38 ± 9.25	0.002
Aromatase inhibitors	Exemestane	31 (47.0%)	32 (68.1%)	0.060
	Letrozole	14 (21.2%)	8 (17%)	
	Anastrozole	21 (31.8%)	7 (14.9%)	
Drink coffee		12 (18.2%)	12 (25.5%)	0.346
Smoking history		1 (1.5%)	0	0.397
Alcohol intake history		2 (3.0%)	1 (2.1%)	0.769
Exercise		27 (40.9%)	14 (29.8%)	0.226
History of fractures		8 (12.1%)	5 (10.6%)	0.808
History of fracture in parents		14 (21.2%)	6 (12.8%)	0.246
FRAX	Major osteoporotic fracture index	3.66 ± 3.39	2.11 ± 1.26	<0.001
	Hip fracture index	0.89 ± 1.76	0.17 ± 0.22	<0.001
WOMAC		34.09 ± 12.32	33 ± 10.36	0.960
M-SACRAH		15.05 ± 8.25	15.83 ± 11.89	0.676

BMI, body mass index; FRAX, Fracture Risk Assessment Tool; WOMAC, Western Ontario and McMaster Universities Osteoarthritis Index; M-SACRAH, Modified Score for the Assessment of Chronic Rheumatoid Affections of the Hands.

**Table 2:** Results of univariate analysis of blood indicators in normal and osteopenia groups.

Variables	Osteopenia group (n = 66)	Normal group (n = 47)	*p*-Value (2-sided)
*White blood cell count (10^9^/L)	4.84 ± 1.00	5.10 ± 1.19	00.226
*Red blood cell count (10^12^/L)	4.50 ± 0.34	4.47 ± 0.42	00.988
*Neutrophil count (10^9^/L)	2.73 ± 0.76	3.02 ± 0.97	00.139
*Lymphocyte count (10^9^/L)	2.17 ± 3.60	1.72 ± 0.50	00.668
*Hemoglobin (g/L)	135.18 ± 8.11	136.13 ± 9.99	00.399
*Platelet count (10^9^/L)	201.02 ± 39.45	206.34 ± 46.72	00.514
^#^Alanine aminotransferase (U/L)	22.17 ± 15.62	20.85 ± 13.26	00.954
^#^Aspartate aminotransferase (U/L)	22.28 ± 7.14	21.73 ± 7.17	00.558
^#^γ-Glutamyl transpeptidase (U/L)	25.38 ± 17.42	27.72 ± 17.42	00.459
^#^Alkaline phosphatase (U/L)	86.27 ± 35.97	77.97 ± 23.90	00.377
^#^Total protein (g/L)	74.11 ± 3.88	73.58 ± 6.08	00.722
^#^Total bilirubin (μmol/L)	13.63 ± 7.57	12.56 ± 5.99	00.193
^$^Creatinine (μmol/L)	54.97 ± 10.47	53.56 ± 15.16	00.947
^$^Blood urea nitrogen (mmol/L)	4.84 ± 1.13	4.71 ± 1.13	00.584
^$^Calcium (mmol/L)	2.42 ± 0.10	2.42 ± 0.11	00.804
**Estradiol (pg/ml)	21.85 ± 92.80	24.05 ± 110.11	00.076
**Follicle-stimulating hormone (IU/L)	33.62 ± 33.31	30.06 ± 34.37	00.192
**Luteinizing hormone (IU/L)	12.07 ± 16.24	10.29 ± 15.27	00.781
**PRL (ng/ml)	172.56 ± 85.30	236.43 ± 122.33	00.012
**Progesterone (ng/ml)	0.82 ± 0.55	0.70 ± 0.32	00.589
**Testosterone (nmol/L)	1.36 ± 2.86	1.26 ± 1.38	00.586
^##^25-Hydroxyvitamin D (nmol/L)	73.21 ± 23.18	68.54 ± 22.43	00.286
^##^β-Isomerized C-telopeptide (pg/ml)	551.51 ± 283.52	641.77 ± 275.78	00.094
^##^Osteocalcin (ng/ml)	23.64 ± 9.56	27.65 ± 10.32	00.025
^##^Growth hormone (μg/L)	1.08 ± 1.39	0.98 ± 1.25	00.566

*represents blood routine test, ^#^represents liver function test, ^$^represents kidney function test, **represents sex hormone level test, and ^##^represents bone metabolism level test.

PRL, prolactin.

### Construction and validation of logistic regression prediction model for AIBL

Six significant risk factors were identified by univariate analysis, including duration of breast cancer, duration of aromatase inhibitor therapy, major osteoporotic fracture index, hip fracture index, PRL, and OC. Subsequently, the Akaike information criterion (AIC) method was used to select independent variables. Multivariate logistic regression analysis revealed that the three relevant variables included in the logistic model were the duration of breast cancer, hip fracture index, and PRL.

**Figure 2:**
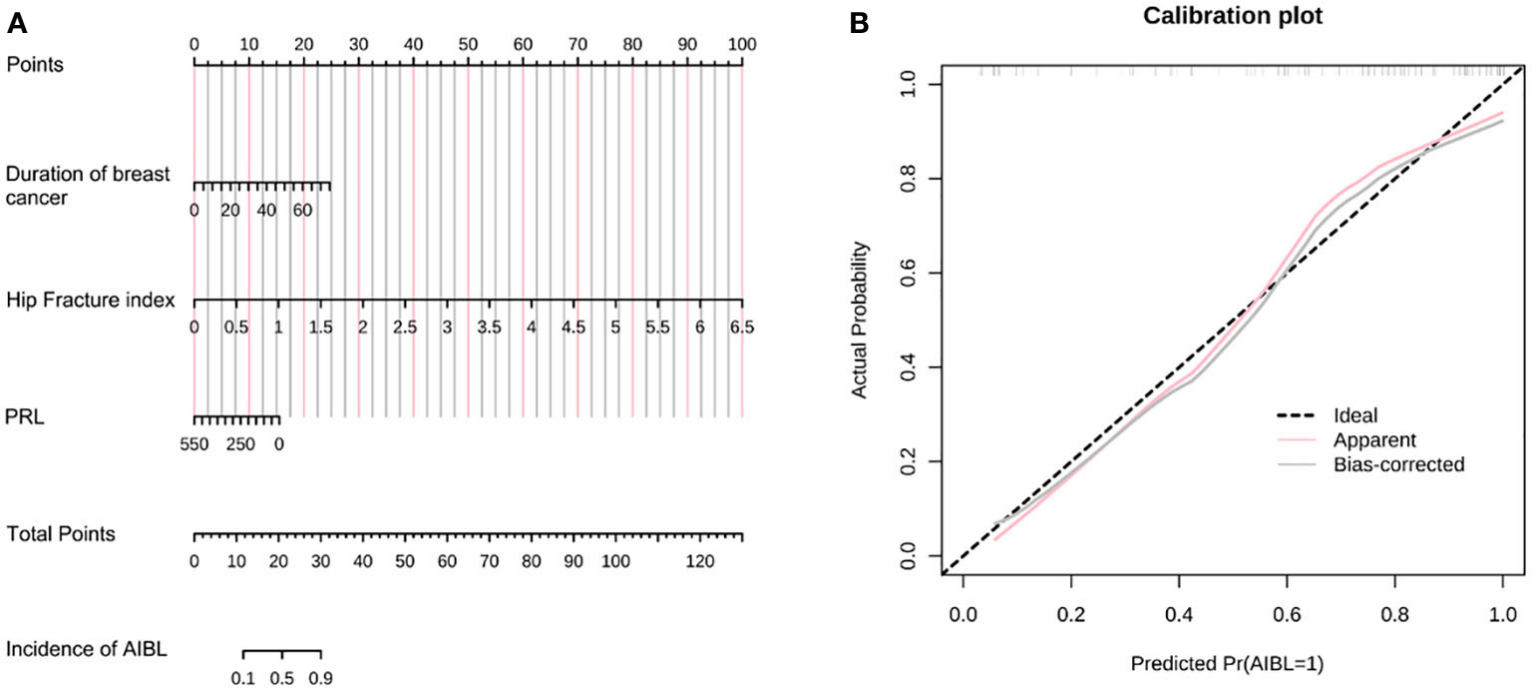
**(A)** The nomogram of AIBL incidence in hormone receptor-positive breast cancer patients using the logistic regression method. **(B)** The calibration curve of the nomogram. AIBL, aromatase inhibitor-associated bone loss.

Among them, the duration of breast cancer and hip fracture index were independent risk factors for AIBL (*p* < 0.05) (**Supplementary Table 1**). The dataset was randomly split into a training set (70%) and a test set (30%). The logistic prediction model was constructed for these three variables in the training set (as shown in **Table 3**), and the corresponding nomogram was drawn (as shown in **Figure 2A**).

**Table 3:** Multivariable logistic regression model with stepwise variable selection.

Variables	*β* coefficient	Z	*p*-Value
Constant	−1.272	−1.042	0.298
Duration of breast cancer	0.079	2.427	0.015
Hip fracture index	3.673	2.919	0.004
PRL	−0.007	−1.826	0.068

PRL, prolactin.

Assuming that the probability of AIBL in patients with hormone receptor-positive breast cancer treated with aromatase inhibitors is P, Logit(P) = −1.272 + 0.079 * (duration of breast cancer) + 3.673 * (hip fracture index) − 0.007 * (PRL). As shown in the table, a longer duration of breast cancer after diagnosis and a higher hip fracture index were associated with a higher incidence of osteopenia. However, patients with higher PRL levels had a lower incidence of osteopenia. The length of the line segment corresponding to each variable in the nomogram represented the degree of influence on the outcome variable (i.e., the occurrence of osteopenia). The corresponding score or category of the variable represents the probability of osteopenia.

**Figure 3:**
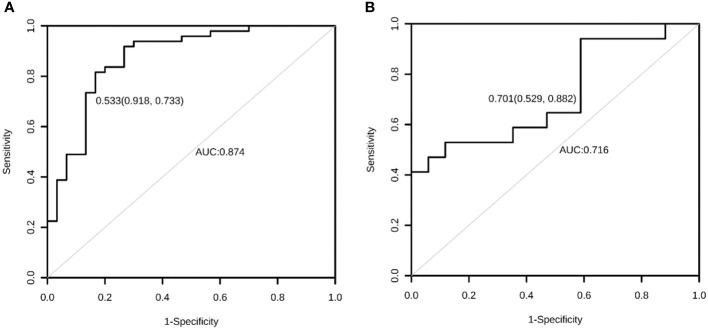
The ROC curve of the AIBL prediction model for the training set **(A)** and the test set **(B)** using the logistic regression method. ROC, receiver operating characteristic; AIBL, aromatase inhibitor-associated bone loss.

Calibration refers to the degree to which the predicted probability of an outcome is consistent with the observed probability. The calibration curve showed that the predicted probability of AIBL in the training set was close to the actual probability when the risk of AIBL was low, while there was an overestimation or underestimation when the actual probability was high (**Figure 2B**). The receiver operating characteristic (ROC) curve was used to reflect the sensitivity and specificity of the prediction model. The ROC curve’s ordinate can measure the sensitivity of the model, and the value on the abscissa is the inverse measure model’s specificity (1 − specificity). The area under the ROC curve (AUC) is usually used as the model’s performance measure. As shown in **Figure 3**, the AUC of the AIBL prediction model based on the logistic regression method was 0.874, the specificity was 0.918, the sensitivity was 0.733, and the cutoff value was 0.533. In the validation set, the corresponding values were 0.716 for AUC, 0.529 for specificity, and 0.882 for sensitivity, and the cutoff value was 0.703. Therefore, the logistic prediction model of AIBL was found to be deficient in terms of calibration and discrimination.

### Construction and validation of the LASSO regression prediction model

**Figure 4:**
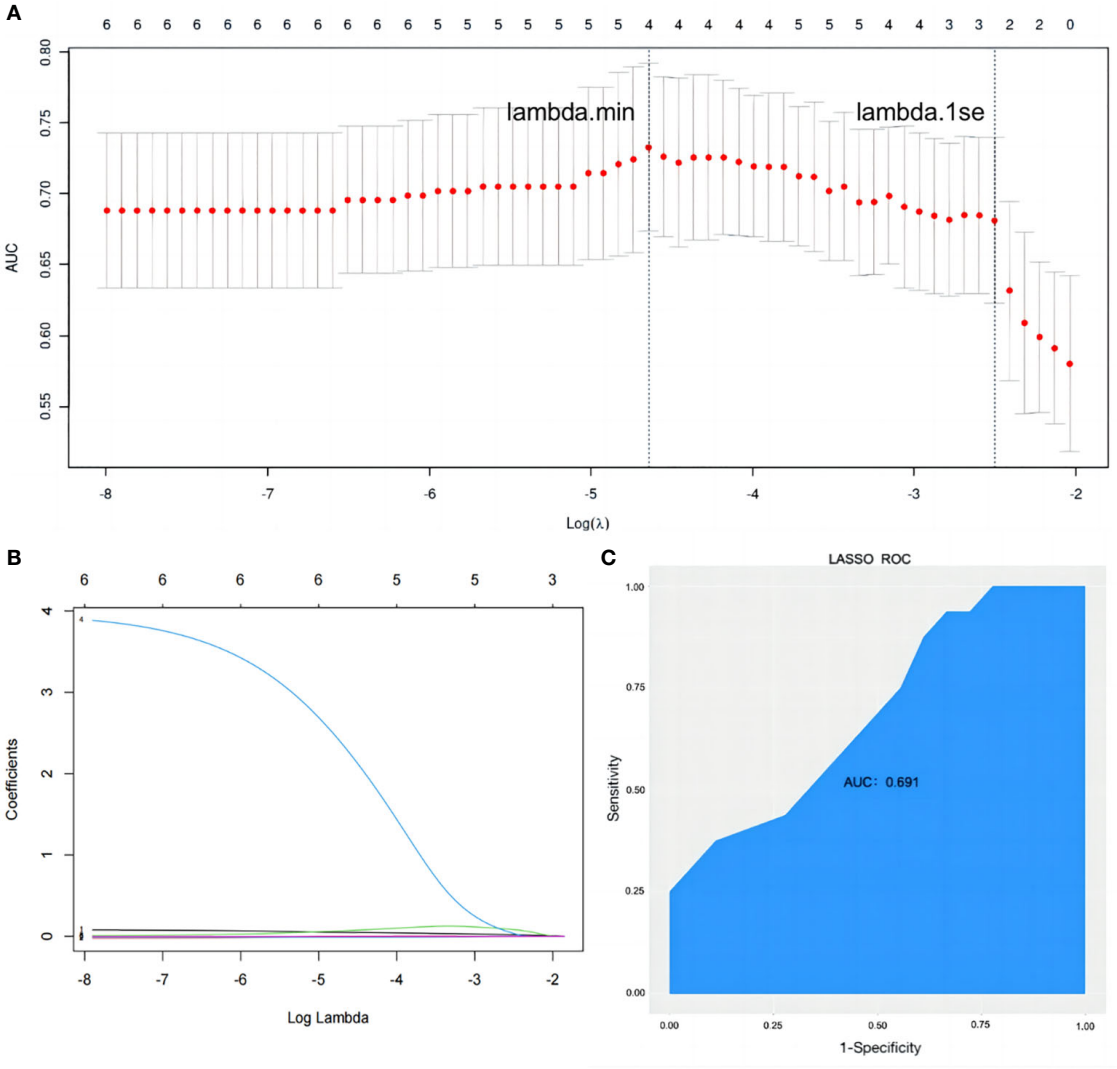
Establishment and validation of a binary outcome prediction model based on LASSO regression. **(A)** In the LASSO model, the optimal parameter (lambda) was selected using a fivefold cross-validation approach. The log(lambda) plot was used to identify lambda.1se, which was used to obtain the included feature factors. The relationship between AUC and log(lambda) was shown. **(B)** LASSO coefficient profiles of the six variables. **(C)** The ROC curve of prediction model. LASSO, least absolute shrinkage and selection operator; AUC, area under the receiver operating characteristic curve; ROC, receiver operating characteristic.

The dependent variable in this study was the occurrence of osteopenia, while the six identified risk factors were considered independent variables in the LASSO regression model. The penalty term coefficient lambda was determined *via* fivefold cross-validation. The results showed that the AUC was maximal when the model was compressed to three variables (**Figure 4A**), and the corresponding lambda was 0.0817. The three variables selected were the duration of breast cancer, hip fracture index, and prolactin, which were consistent with the results of the logistic model. The model coefficients corresponding to the three variables are shown in **Supplementary Table 2**. The degree of compression of the six variables under different penalty parameter lambda is illustrated in **Figure 4B**. In classification models, the AUC value is commonly used as the evaluation index. The expression of the LASSO model on the test set was poor, with an AUC value of only 0.691 (**Figure 4C**).

### Construction and validation of a prediction model using XGBoost machine learning algorithm

XGBoost was developed by Tianqi Chen in 2016 and is based on the idea of building a basic learner in the training set (8), adjusting the sample distribution according to the results of the basic learner and repeating this process until the number of basic learners reaches the set value. The XGBoost algorithm has been widely used in disease risk prediction in medical research (15–17).

**Figure 5:**
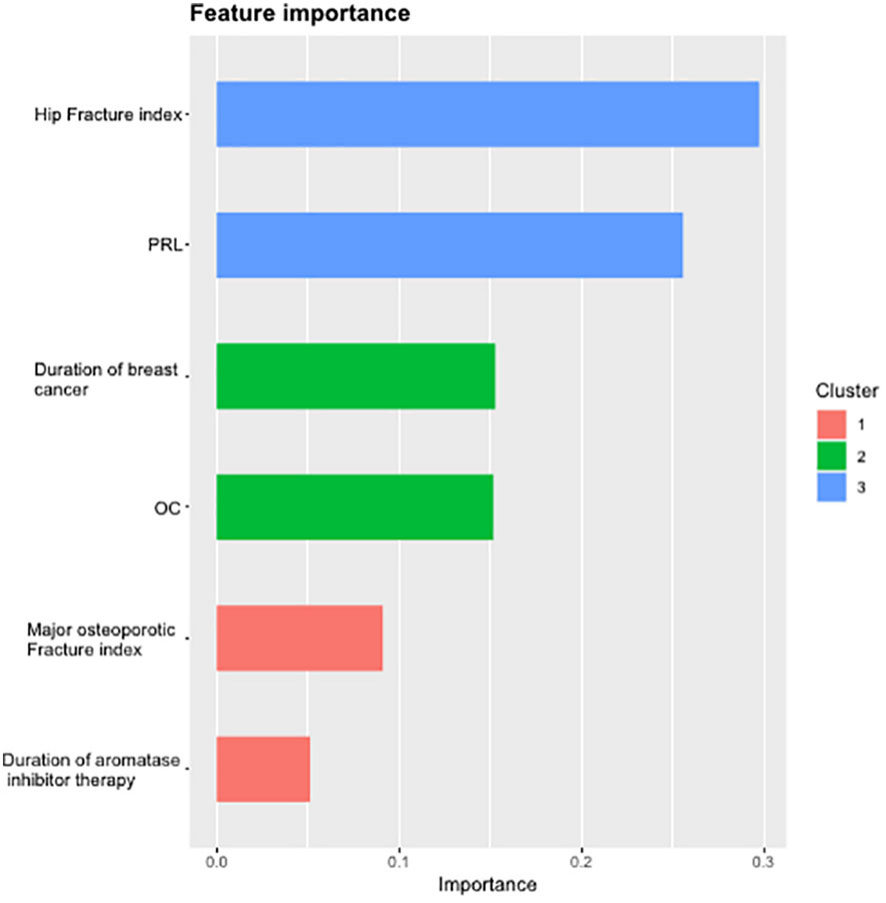
Importance ranking diagram of variables in AIBL prediction model. AIBL, aromatase inhibitor-associated bone loss.

The dataset of 113 subjects was randomly divided into training and test sets in a 7:3 ratio. The XGBoost prediction model was constructed by adjusting each parameter, and the importance of the six variables included in the AIBL model was calculated and arranged (as shown in **Table 4**; **Figure 5**). Hip fracture index and prolactin were found to be the two most important factors, accounting for more than half of the proportion, followed by the duration of breast cancer and osteocalcin level, which accounted for one-third. Major osteoporotic fracture index and aromatase inhibitor treatment time accounted for more than 5%. The sum of the importance ratios of all features was 1.

**Table 4:** The ranking table of the importance of the XGBoost algorithm for the six variables in the AIBL prediction model.

Variables	Gain	Cover	Frequency	Importance
Hip fracture index	0.30	0.20	0.13	0.30
PRL	0.26	0.22	0.25	0.26
Duration of breast cancer	0.15	0.14	0.12	0.15
OC	0.15	0.22	0.23	0.15
Major osteoporotic fracture index	0.09	0.16	0.19	0.09
Duration of aromatase inhibitor therapy	0.05	0.05	0.09	0.05

XGBoost, eXtreme gradient boosting; AIBL, aromatase inhibitor-associated bone loss; PRL, prolactin; OC, osteocalcin.

**Figure 6:**
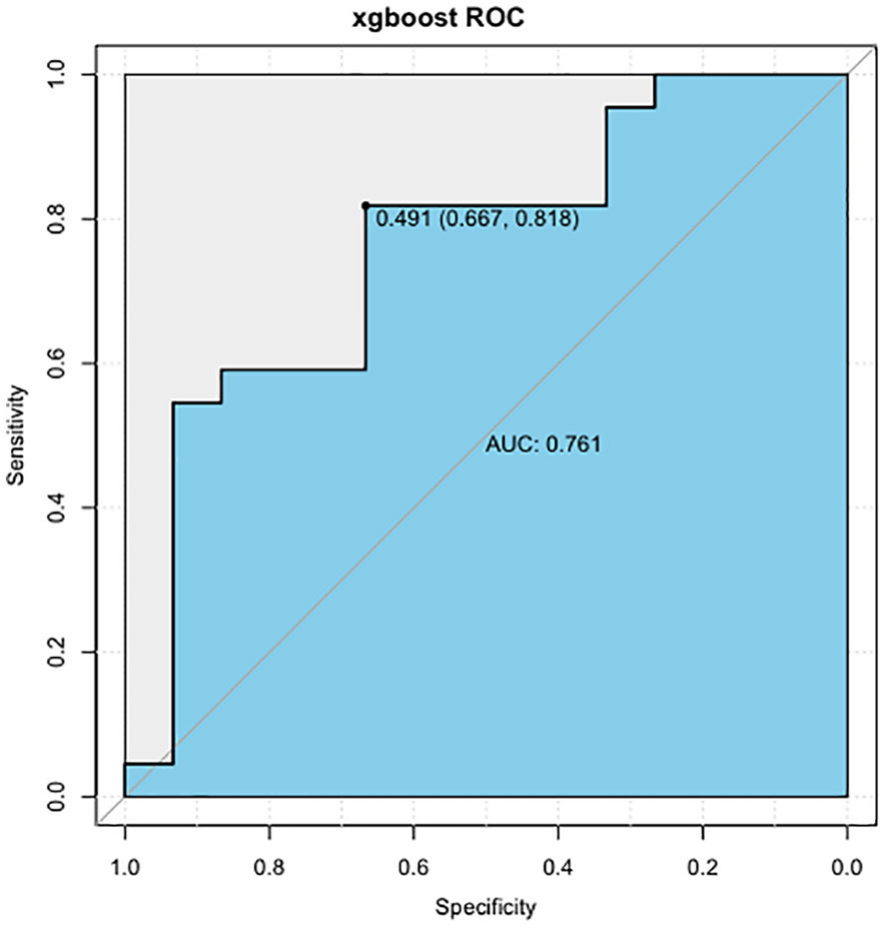
ROC curve of AIBL prediction model constructed by XGBoost algorithm. ROC, receiver operating characteristic; AIBL, aromatase inhibitor-associated bone loss; XGBoost, eXtreme gradient boosting.

In summary, the AIBL prediction model constructed by the XGBoost machine learning algorithm has demonstrated the importance of each characteristic variable, and no irrelevant information was included. The random split validation method was employed for internal validation of this part of the XGBoost machine learning prediction model. The test set data were incorporated into the XGBoost prediction model while adjusting the parameters: the maximum depth of the tree was set to 6, the learning rate to 0.5, and the maximum number of iterations to 25, and the other parameters were set to default. The ROC curve (**Figure 6**) was generated, and the AUC was found to be 0.761, with a specificity of 0.667, a sensitivity of 0.818, and a cutoff value of 0.491. These results indicate that the XGBoost model exhibits excellent predictive performance.

### Summary

This study included a total of 113 eligible patients of whom 66 had osteopenia. A set of 48 candidate predictors, comprising basic patient information, breast cancer diagnosis and treatment, and three scales of FRAX, WOMAC, and M-SACRAH, involving 23 variables, as well as 25 blood index tests were identified. With the use of logistic, LASSO, and XGBoost algorithms, three prediction models were constructed and validated to predict the incidence of AIBL in hormone receptor-positive breast cancer patients. The logistic and LASSO regression models included three predictive factors, namely, the duration of breast cancer, hip fracture index, and prolactin. The performance of the three models was evaluated using the area under the ROC curve, with the XGBoost model demonstrating superior performance.

## Discussion

The mechanism of AIBL in hormone receptor-positive breast cancer patients is analogous to that of postmenopausal women with osteoporosis, where a significant decrease in estrogen levels leads to bone loss. However, AIBL patients are affected by various factors, such as OFS treatment, radiotherapy, and chemotherapy, in addition to AI treatment. The screening tools for osteoporosis, such as OSTA (18) and SCORE (19), which are applicable to all women, mainly consider recognized high-risk factors such as age, body mass index (BMI), and fracture history. These tools cannot comprehensively screen high-risk factors in the AIBL group. Therefore, the AIBL model constructed in this study serves as a supplement to current screening tools in the field of osteoporosis. The primary target of the prediction model is the high-risk factors of AIBL in hormone receptor-positive breast cancer patients. This model aims to provide technical support for early intervention and further refine screening for this high-risk group.

The hip fracture index is a significant high-risk factor for AIBL in both models and is consistent with previous research (11). PRL is secreted by the anterior pituitary gland, and studies have shown that women with hyperprolactinemia and prolactinomas have a higher incidence of vertebral fractures when compared to the normal population (20). Another study suggests that an increase in normal PRL levels may have a positive effect on BMD in patients with type 2 diabetes mellitus (T2DM) rather than a significant increase in PRL (21). Our study indicates that higher levels of PRL are negatively associated with the development of AIBL and may serve as protective factors. However, the study sample size was inadequate to provide a specific numerical range for the protective effect of PRL. Furthermore, there is a lack of *in vivo* or *in vitro* animal experiments to further elucidate the underlying mechanisms.

A meta-analysis of screening tools for osteoporosis (22) highlighted a common issue that the sensitivity was usually close to or above 90% at a specific threshold, the specificity was often below 50%, and the AUC values were generally between 0.5 and 0.8. In contrast, the XGBoost model developed in this study achieved a sensitivity of 66.7% and a specificity of 81.8% at a threshold of 0.491, with an AUC of 0.761. The AIBL risk prediction model incorporates blood indicators, which may be more expensive than medical history collection but can avoid the bias of subjective reporting and provide more reliable information. Moreover, the blood indicators used in the model are common in China and are more accessible than BMD testing. Using the risk prediction model to screen patients who have recently undergone blood tests can prompt the diagnosis of osteopenia by BMD testing. The AIBL prediction model can also be used to screen patients with metal implants who cannot undergo BMD testing to reduce the rate of missed diagnosis. Although the three risk prediction models developed in this study may not be as simple and economical as the FARX screening tool, they can screen patients with a high risk of bone loss based on the existing blood test results without additional economic cost. Despite routine calcium supplementation during AI treatment, more than half of patients still develop osteopenia or osteoporosis, suggesting that calcium supplementation alone may not meet the body’s needs. Increasing the dose of calcium supplementation, adding vitamin D, or even bisphosphonates may be necessary for high-risk AIBL patients. In conclusion, the risk prediction models developed in this study using traditional logistic regression, LASSO regression, and XGBoost algorithm have clinical significance and practical application value, with the XGBoost algorithm demonstrating better performance.

In recent years, machine learning has been increasingly applied in the medical field, including in the development of bone loss risk prediction models. This study utilized the XGBoost algorithm to construct an AIBL prediction model, which complements the traditional logistic and LASSO algorithms and provides a fine division of the applicable population for bone loss. XGBoost machine learning has demonstrated superior performance in dealing with multiple complex variables and non-linear problems compared to traditional logistic regression and LASSO machine learning algorithms (15). However, the small number of research subjects and screening variables in this study limits the prediction performance of XGBoost. Future studies should continue to collect patients who meet the criteria and expand the database to observe the powerful performance of the XGBoost algorithm in processing high-dimensional data in building a risk prediction model. The AIBL prediction model developed in this study involves the cost of blood index testing, which may make it difficult to obtain information and promote implementation. Additionally, the lack of external validation raises concerns about the model’s suitability for large-scale use in the real world. Future research should focus on the derivation and improvement of the AIBL prediction model for early warning of osteoporosis, specifically screening patients with osteoporosis according to blood biochemical indicators (T < −2.5). This population is at higher risk of fracture and experiences more noticeable arthralgia. The warning tool can help diagnose patients in a timely manner, adopt corresponding treatment, improve compliance with AI endocrine drugs, and successfully complete the full course of breast cancer treatment, which is the main goal of this study.

## Data availability statement

The original contributions presented in this study are included in the article/**Supplementary Material**. Further inquiries can be directed to the corresponding authors.

## Ethics statement

The Medical Ethics Committee of Longhua Hospital affiliated with the Shanghai University of Traditional Chinese Medicine approved this research.

## Author contributions

MC and YZ: conceptualization, formal analysis, data curation, and visualization. MC: writing—original draft. YZ and LJ: validation, visualization, and writing—review and editing. YY: methodology. HC, TM and BH: investigation, validation, and data curation. JW and MY: supervision, project administration, and funding acquisition. All authors contributed to the article and approved the submitted version.
