# Supplementary material for: Construction and validation of a risk prediction model for aromatase inhibitor-associated bone loss
Source: Front Oncol. 2023 Apr 27;13:1182792. doi: 10.3389/fonc.2023.1182792 (PMC10174287; doi:10.3389/fonc.2023.1182792)
Supplement: Supplementary file 1 [file Table_1.docx]

Supplementary Table 1 Results of multivariate analysis of variables in the study population

| Variables | Z | *P* value | OR[95%CI] |
| --- | --- | --- | --- |
| Duration of breast cancer | 2.073 | 0.04 | 1.08[1.00,1.17] |
| Duration of aromatase inhibitor therapy | -0.673 | 0.50 | 0.97[0.90,1.06] |
| Major Osteoporotic Fracture Index | 0.025 | 0.98 | 1.01[0.68,1.48] |
| Hip Fracture index | 2.934 | 0.003 | 53.35[3.75,759.89] |
| OC | -0.429 | 0.67 | 0.99[0.94,1.04] |
| PRL | -1.638 | 0.10 | 0.99[0.99,1.00] |
