# Supplementary material for: Construction and validation of a risk prediction model for aromatase inhibitor-associated bone loss
Source: Front Oncol. 2023 Apr 27;13:1182792. doi: 10.3389/fonc.2023.1182792 (PMC10174287; doi:10.3389/fonc.2023.1182792)
Supplement: Supplementary file 2 [file Table_2.docx]

Supplementary Table 2 Coefficients of variables in the model

| Variables | coef |
| --- | --- |
| Duration of breast cancer | 0.002 |
| Hip Fracture index | 0.110 |
| PRL | -0.002 |
